# Body-size-dependent effects of landscape-level resource energetics on pollinator abundance in woodland remnants

**DOI:** 10.1098/rspb.2023.2771

**Published:** 2024-06-12

**Authors:** Juliana Pille Arnold, Jason M. Tylianakis, Mark V. Murphy, Gregory R. Cawthray, Bruce L. Webber, Raphael K. Didham

**Affiliations:** ^1^ School of Biological Sciences, The University of Western Australia, Crawley, Western Australia, 6009, Australia; ^2^ CSIRO Health & Biosecurity, Centre for Environment and Life Sciences, Floreat, Western Australia, 6014, Australia; ^3^ Bioprotection Aotearoa, School of Biological Sciences, University of Canterbury, Christchurch, New Zealand

**Keywords:** body size, floral resources, mutualistic network, land use change, landscape scale, pollinator decline

## Abstract

Land use change alters floral resource availability, thereby contributing to declines in important pollinators. However, the severity of land use impact varies by species, influenced by factors such as dispersal ability and resource specialization, both of which can correlate with body size. Here. we test whether floral resource availability in the surrounding landscape (the ‘matrix’) influences bee species’ abundance in isolated remnant woodlands, and whether this effect varies with body size. We sampled quantitative flower-visitation networks within woodland remnants and quantified floral energy resources (nectar and pollen calories) available to each bee species both within the woodland and the matrix. Bee abundance in woodland increased with floral energy resources in the surrounding matrix, with strongest effects on larger-bodied species. Our findings suggest important but size-dependent effects of declining matrix floral resources on the persistence of bees in remnant woodlands, highlighting the need to incorporate landscape-level floral resources in conservation planning for pollinators in threatened natural habitats.

## 1. Introduction

Land use change threatens biodiversity and ecosystem functioning by altering the abundance and distribution of organisms [1,2]. Projected increases in habitat loss and fragmentation [3] are expected to drive further biodiversity decline, while novel ecological barriers to dispersal will increase the isolation of remnant habitat patches [4]. Whether this remnant habitat will be sufficient to prevent further biodiversity loss may depend partly on whether the surrounding landscape of natural and modified habitat (the ‘matrix’) is of sufficient quality to overcome these barriers and support remnant populations [4–6].

Among the multiple pressures that land use change imposes on pollinators [1,7,8], floral resource limitation due to a decline in preferred flowering species or increases in non-suitable floral resources, such as non-native plants [9,10], strongly contribute to pollinator declines [8,11,12], particularly of larger bee species [13]. Moreover, parallel declines in insect-pollinated plant species following declines of their pollinators [14] highlight strong interdependencies that mediate their joint responses to land use change [15].

Recent strategies to mitigate pollinator declines have thus far focused on restoring natural or semi-natural habitats [16–18] or enhancing floral resources for pollinators, such as through planting of wildflower strips adjacent to agricultural fields [19–21]. While expansion of natural habitat into modified landscapes is rare, the management of the surrounding matrix can help to mitigate the impacts of land use change on pollinators and pollination services [22,23]. More generally though, the moderating role of floral resource availability in the surrounding matrix on pollinator species within natural remnants remains relatively poorly understood (but see studies by [19,24–26] in agricultural landscapes).

The floral resource requirements of pollinators depend on their body size [27]. For instance, in larger vertebrate animals (e.g. nectar-feeding birds) metabolic demand increases with body mass so that large individuals require greater total energy per capita, but this translates to less energy per gram of body mass (hypometric scaling) [28]. In insects, however, Duell *et al*. (2022) [29] found that the relationship between flight metabolic rate and body mass does not change in a simple uniform manner, but scales hypermetrically in smaller insects (< 58 mg body mass), and hypometrically in the larger size range. Therefore, pollinators tend to visit flowers with nectar caloric value that correlates with their body size [28,30–32], such that the greater energetic gain relative to flight costs of flower visitation results in increased foraging efficiency [33]. In this way, body size can also mediate niche partitioning among co-occurring species [28,33]. Body size also affects flight efficiency [34], and larger bees forage disproportionally further than smaller bees [35], enabling them to better exploit patchy or scattered resources. Consequently, foraging distance in large-bodied bees varies as a function of landscape context, increasing with resource scarcity [36,37]. Thus, declines in floral resources due to land use change can affect large-bodied and small-bodied bees differently [38,39], but this difference will likely depend on the ability of different species to exploit different plant partners [40]. Importantly, body size of pollinators can be an important trait for determining the number and identity of interaction partners [41], such that resource requirements, the ability to forage across the landscape, and the ability to interact with a diverse set of plant resources may all become interdependent via their relationship with body size [42]. Yet, the few previous attempts to assess the importance of floral resources for pollinators at wider scales than the local-level have used coarse estimates of floral resources in different land-use types [24,26,43–45] or in an entire floral community [46] rather than a detailed assessment of nectar and pollen resources for partner plant species as replicates for understanding energetics, thereby obscuring any differences in the rewards available to different pollinator species, and how this depends on their body size.

Here, we estimate floral resource energy available to pollinators at the landscape-level (in remnant woodland patches and their surrounding mosaic landscape matrix) by directly measuring both nectar and pollen resources from flowers of partner plant species in a biodiversity hotspot in southwest Western Australia. We focus on bees (Hymenoptera: Apoidea: Anthophila), a highly diverse key pollinator group that depends on flowers for nutrition [47], and sample quantitative flower–bee visitation networks from the remnant woodlands. We address the following hypotheses: (i) the abundance of a bee species in remnant woodland patches will increase with increasing energetic availability of its partner species’ floral resources (nectar + pollen) within remnants and in the surrounding landscape and (ii) small-bodied bee species (which have lower energetic requirements and shorter foraging ranges) will be more influenced by within-patch floral resources, while larger species will benefit from resources at both patch- and landscape-level.

## 2. Methods

We recorded plant–pollinator interactions within remnants to determine the plant species visited by each pollinator species, then quantified the availability of these partner species in the surrounding matrix. We also used pollen and nectar samples to quantify the energy available per flower for each plant species and combined this with landscape data to infer the landscape-level availability of energy for each pollinator species, linking it to body size data. We describe each of these steps below.

### (a) Study system

**Figure 1.**
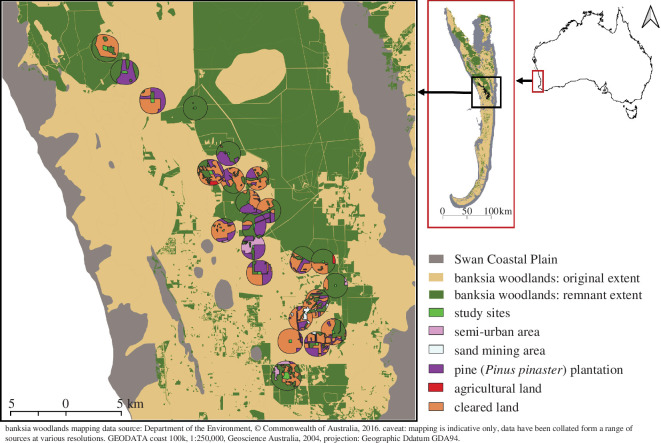
Locations of the 23 banksia woodland study sites in a 152 km^2^ area of the Gnangara-Moore River State Forest, Swan Coastal Plain, Western Australia (31.77 °S, 115.93 °E). Sites are coloured neon green and 1 km radius buffer zones around each site are shown, with land cover classified according to land use. Also shown is the original (pre-European settlement) and remnant extent of banksia woodland in the region.

We conducted the study in a mosaic landscape of remnant banksia woodland on the Swan Coastal Plain, located in the Mediterranean-climate Southwest Australia biodiversity hotspot [48]. Banksia woodland is recognized as a threatened ecological community in Australia under the Environmental Protection and Biodiversity Act 1999 [49]. The remaining native vegetation consists of small remnants subject to increasing pressure from anthropogenic land uses in the surrounding matrix (figure 1).

We surveyed 23 woodland remnants located within a 152 km^2^ area of the Gnangara-Moore River State Forest, Western Australia (−31.85° N, 115.92° E; −31.54° N, 115.73° E) (figure 1). The 23 remnants varied in size from 1 to 31 hectares, and site suitability was ground-truthed to ensure that remnants had a relatively consistent level of habitat quality (i.e. consistently low level of habitat degradation, including minimal signs of human disturbance, minimal presence of non-native plants and minimal evidence of *Phytophthora* dieback in native vegetation), and no unintended covariance between patch attributes and any underlying ‘distance from coast’ (east–west) or latitudinal (north–south) gradients within the study area. The selection of sites was also based on variation in surrounding land uses, where we used the proportion of banksia woodland cover in the landscape as a criterion (in lieu of any prior knowledge of the availability of floral resources in other land uses) that might influence flower-visiting insects. Land-use types in the matrix surrounding the selected remnants consisted of a mosaic of native vegetation (distinct patches of banksia woodland) and anthropogenic land uses, including agricultural land, non-native pine plantation (*Pinus pinaster* Aiton), cleared land covered by early successional regeneration, semi-urban areas and commercial sand mining sites (figure 1).

### (b) Plant–pollinator interaction network sampling

We recorded bee (Hymenoptera: Apoidea: Anthophila) visits to flowers in each of the 23 woodland remnants using four 50 m × 4 m parallel belt transects spaced at 20 m intervals. We subdivided each belt transect into 10 m sections and spent 15 min per section hand-collecting bees alighting on flowers up to 2 m from the ground using a sweep net and recording each plant–pollinator association. We conducted sampling during the spring season (September to November) in 2015 and 2016, only during standardized weather conditions (calm, sunny weather), surveying transects in random order and at random times across sites during the peak activity period (09.00 to 16.00) to control for temporal variation in pollinator assemblage composition and flower visitation throughout the day (5–10 h survey effort per site as some sites were not able to be sampled in 2015 owing to a shorter flowering season caused by higher than average temperatures; 195 h total effort across the study). We preserved bee specimens in 70% (v/v) ethanol, which were then pinned and sorted to taxonomic family, morphospecies, and subsequently species (where possible).

### (c) Bee body mass estimation

We measured bee body length (mm) along the lateral side of each specimen (total length from the point of antennal insertion to the terminal abdominal tergite) using a digital calliper under a stereo microscope. We used a predictive model that uses allometric scaling to predict bee body mass (dry mass, mg) as a function of body length, using the bee morphological trait dataset in the R package *pollimetry* [50]. The model estimates log(body mass) based on measured values of log(body length), taxonomic family, and sex. We included taxonomic family as a predictor in the model to account for phylogenetic relatedness, since it has been shown to be predictively equivalent to phylogenetic data in allometric scaling relationships in bees [51]. We incorporated sex to account for sexual size dimorphism (SSD), since the majority of bee species exhibit female‐biased SSD (i.e. females are larger than males) [52]. We measured up to 10 females and 10 males of each species, where specimens were available. Of the 53 bee species, we measured a total of 435 specimens (287 females and 148 males; mean ± s.d. number of measured specimens per species 8.2 ± 4.6; electronic supplementary material, table S1) to estimate body mass (as dry mass, mg). Body mass ranged from 0.45 ± 0.03 mg (mean ± s.e.) for *Pachyprosopis* sp.1 (Colletidae) to 79.80 ± 5.36 mg (mean ± s.e.) for *Megachile semiluctuosa* (Megachilidae), with an average body mass across all species of 18.33 ± 0.92 mg (mean ± s.e.; electronic supplementary material, table S1).

### (d) Landscape flower density assessment

We assessed floral resource availability by estimating flower density—the number of open flowers per unit area of land-use type—of the pollinator-specific plant species (i.e. network interaction partners of each bee species) found in representative transects in banksia woodland remnants and in the surrounding matrix land-use types, and subsequently measuring nectar and pollen amount per flower.

In the remnants, we carried out flower density surveys in two parallel 50 m × 4 m belt transects, spaced 20 m apart. It was not possible to survey all sites at the same time within the brief peak flowering period (October 2016); therefore, we used two approaches: direct counts of flower density (number of flowers per species in each transect was directly counted where possible or estimated where flower abundance was particularly high), and indirect photographic surveys (overhead photographs of the vegetation were taken every 3 s while simultaneously recording video). The photographs and videos were subsequently analysed in the laboratory by a trained botanist to estimate flower density.

For the surrounding matrix, we quantified flower density within 1 km radius buffer zones (landscapes) around the perimeter of each woodland remnant (figure 1). We classified land-use types manually using high-resolution aerial imagery (10 cm per pixel; Nearmap™, Perth, Australia) and information from local land surveys in a geographic information system (GIS) in QGIS 3.2.2 ‘Bonn’ [53]. Land cover was dominated by three land-use classes: pine plantation, cleared land and banksia woodlands, representing 97% of total land cover, so flower density was only estimated in these classes. We surveyed flower density in the matrix using a total of 22 belt transects (30 m × 4 m), with 15 in cleared land and seven in pine plantations. Transects were surveyed across the study area at random locations, starting at a random point and oriented in a north–south direction. Relative sampling effort was not directly proportional to the land cover of cleared land (52%) versus pine plantation (10%), leading to a slight proportional over-representation of sampling in pine plantations. However, we felt it was important to have a reasonable minimum number of transects to provide robust characterization of flower resources in pine plantations, but we did not have the financial or logistical ability to achieve five times this effort in cleared land uses. Banksia woodland in the matrix was the same land-use type as the selected remnants, with similar habitat quality, therefore, we did not do any additional surveys in this land-use type in the matrix. For all matrix transects, we directly estimated flower density in the field using the same procedures as for remnant transects.

### (e) Nectar collection and analysis

We were able to collect nectar samples for 33 of the 56 plant species within our plant–pollinator networks (as part of a larger collection of 70 common plant species in the study area). We collected nectar from four to seven (median = 5.5) flowers per species, incorporating intraspecific and spatiotemporal variation in nectar properties where possible [43,54]. Because nectar secretion rate varies through the day [55], and because flower visitors can deplete available nectar, we bagged unopened flower buds *in situ* for 24 h using a 0.5 mm × 0.5 mm organza fabric mesh bag and collected the accumulated nectar in each flower (standing crop) the following day between 08.00 and 12.00 using one of three protocols adapted from [44,56], depending on the flower type: (i) sampling directly using microcapillary tubes (0.5–10 μl capacity, Drummond Scientific Co., USA), (ii) rinsing the flower nectaries with 1 µl of distilled water using a micropipette and collecting the diluted nectar solution with a microcapillary tube, or (iii) washing the entire flower in a known volume of distilled water in a plastic tube. We measured nectar volume in microcapillary tubes before transferring the nectar solution into insert tubes for high-pressure liquid chromatography analysis (HPLC, 600E pump, 717 plus auto-injector, 2996 Photodiode array detector Waters Corporation, Milford, USA; Alltech Evaporative Light Scattering Detector (ELSD 500), Grace Materials Technologies, Deerfield, IL, USA) of the three dominant sugars fructose, glucose and sucrose [57].

### (f) Pollen collection and analysis

We were able to collect pollen samples for 36 of the 56 plant species within networks, using at least three flowers (3–585, median = 10) from different plant individuals per species. Flowers were collected as buds that were about to open, stored individually in vials and dried in an oven at 35°C for 48 h, before being stored longer term in labelled screw cap vials in a dark and cool cupboard until pollen removal for analysis. Before pollen removal, we placed flowers back in the oven at 35°C for at least 5 h to eliminate any humidity absorbed during storage. For each species, we used a 1.5 ml Eppendorf vial to store the pollen during the process, which was weighed on a four-decimal place electronic analytical balance with a draft shield (Mettler Toledo AG245, repeatability = 0.1 mg) to record initial weight. We removed pollen from anthers under a stereomicroscope, separating anther tissues using a small sieve (Ø8 mm, 30 mm aluminium tube, 90 µm mesh) attached to the opening of the Eppendorf vial, using a combination of fine brushes and a customized air blow device to direct and force the pollen through the sieve into the vial. We repeated the process until all pollen was removed from anthers, and on the rare occasions that debris was present, we removed it with fine forceps. We weighed the Eppendorf vial again and recorded the difference from the initial weight (i.e. the pollen weight per flower). The amount of pollen per flower was averaged by dividing the total amount of pollen removed by the total number of flowers sampled per species. We measured the pollen energy content of seven species (2–9 samples per species, electronic supplementary material, table S2) to determine an average pollen energy content [32] for plant species in our study area. Pollen samples removed from flowers (approx. 10 mg) were dried in an oven at 35°C for 48 h and compressed into tablets using a tablet press. We used a bomb calorimeter (IKA C1 Compact Calorimeter, IKA^®^-Werke GmbH & Co. KG, Staufen, Germany, temperature measure resolution = 0.0001 K) to determine pollen calorific content. We added 10 drops of paraffin oil (IKA Werke Paraffin Oil C17, Gross Cal Value 46322 J g^−1^, RSD 0.056%) to each tablet as a combustion aid.

### (g) Estimation of nectar and pollen energy per flower

For each of the three dominant sugars, glucose, fructose and sucrose [57], we estimated nectar energy per flower as the mass of each sugar per flower multiplied by its energy content (cal mg^−1^). We calculated energy content for each sugar by dividing the heat of combustion (Δ*Hc*°) of the sugar by its molecular weight (sourced from [58]; electronic supplementary material, table S3). Then, we summed the energy content of each sugar per flower, giving a total energy per flower. Finally, we calculated the mean nectar energy per flower (produced over a 24 h period) per species. We performed calculations of pollen energy per flower using the average pollen amount per flower (mg) per species. We multiplied the mean pollen weight per species by the mean pollen calorific content across all species analysed (4.11 ± 0.15 cal mg^−1^, mean ± s.e., electronic supplementary material, table S2). For the species that had no nectar sampled, we estimated sugar mass values using data recorded for closely related species in our dataset (i.e. from the same genus or family or data from the literature; electronic supplementary material, table S4). We used the same approach for missing pollen mass values. We calculated the total energy per flower per species by summing the mean nectar and pollen energy per flower.

### (h) Construction of plant–pollinator interaction networks

We performed all data analyses in R version 4.3.1 [59]. We constructed quantitative interaction matrices representing plant–pollinator networks for each remnant woodland (*n* = 23), using the number of interaction events recorded between bee species (rows) and flowering plant species (columns). We also constructed a global ‘metaweb’ by pooling all observed interactions between bees and plants across all sites (electronic supplementary material, figure S1). The metaweb provides information on the regional species pool and interactions of co-occurring species, representing a more complete source of information on potential plant resources used by bee species.

### (i) Scaling-up energy from individual flowers to landscape scales

We multiplied the mean nectar and pollen energy per flower per species by the total number of flowers per species per unit area (flower density m^−2^) in each land-use type, extrapolating to the weighted total area of each land-use type in the landscape. For all Asteraceae species, energy was assessed per flower head (capitulum), while for *Banksia attenuata*, *Banksia sessilis* var. *sessilis* and *Xanthorrhoea preissii*, it was assessed per inflorescence using the average number of open flowers per inflorescence. For 13 (23.2 %) plant species recorded in the plant–pollinator networks in remnants but not recorded in any of the representative transects surveyed (due to rarity or aggregated distributions), we assigned a nominal flower density value of half the smallest recorded value of any other plant species in each of the different land uses. This approach recognizes that the plant species does occur in the region but at a very low occurrence in all land-use classes.

We used recorded plant resource preference data from the regional metaweb of all observed interactions between bees and partner plants (electronic supplementary material, figure S1) to estimate the availability of floral resource energy for each bee species within each remnant woodland and in the surrounding land uses by calculating the total amount of floral energy per unit area in each habitat type (flower density per unit area multiplied by the total energy of nectar and pollen per flower). Lastly, we multiplied this total energy by the area of each remnant and by the total area of matrix land uses in the surrounding 1 km radius landscape (figure 1), in order to generate the amount of floral resource energy of plants available for each bee species at the local- and landscape-scale.

### (j) Statistical analyses

We fitted generalized linear mixed effects models (GLMMs) to test the effects of both remnant patch and surrounding landscape floral resource energy availability on the local (within-patch) bee abundance (i.e. the total number of individuals per species) for bee species varying in log-transformed body size. Since there were many instances where zero individuals were recorded for bee species, we followed the approach of Brooks *et al*. (2017) [60], fitting a set of mixed models with different error structures (Poisson, negative binomial and Conway–Maxwell–Poisson) with and without zero-inflation using the R package *glmmTMB* [61]. The most appropriate error distribution for the data was then selected by comparing models using Akaike information criteria (AIC) with the ‘aictab()’ function in the package *AICcmodavg* [62]. Of the models considered, the negative binomial model without zero inflation was the most parsimonious.

Prior to model fitting, all predictors were mean-centred and scaled to two standard deviations using the package *arm* [63], in order to improve the interpretation of model estimates and avoid model convergence issues owing to differences in predictor scales [64]. We specified the natural logarithm of body mass (mg) as a fixed continuous predictor and included its interaction with patch- and landscape-level floral resource energy (along with these main effects). Since patch area could drive increases in bee abundance for reasons other than floral resource availability, potential confounding effects of patch area (Ha) were accounted for by including it as a fixed covariate. We included sampling effort (log-transformed number of sampling hours) as a model offset to account for variation in survey time per site. Species name was included as a random intercept to account for species-specific variation in bee abundance and the non-independence of multiple abundance records for each bee species. We checked for excessive multicollinearity among predictor variables by calculating the variance inflation factor (VIF) for all fixed predictors, retaining those with a VIF value below 3.0 [65] (no predictor variables were removed from the model).

We performed model selection using a multi-model inference approach to determine the minimum adequate model(s) that best reproduces the data [66,67], starting with a full (global) model containing all predictors. This approach reflects our multiple hypotheses of interest, and in particular, whether there is sufficient evidence for the effects of both small-bodied bee species being more influenced by within-patch floral resources and large-bodied bees being more influenced by landscape-level resources or whether a simpler model with only one (or neither) of these effects is more parsimonious. To do this, we compared competing models consisting of all possible combinations of predictors using AIC values corrected for small sample size (AICc), using the function ‘*AICcmodavg*::aictab()*’* [62]. Within the top model set (i.e. models with lower AICc values), the most parsimonious model (i.e. the model with the fewest fitted parameters) was considered to be the ‘best model’, but we also present results from other models within two AICc units of this model for reference [66] (electronic supplementary material, table S7). Subsequently, we used the function ‘*performance*::r2_nakagawa()’ [68] to calculate the coefficient of determination for fixed effects only (marginal; R^2^_GLMM m_) and incorporated both fixed and random effects (conditional; R^2^_GLMM c_) in order to provide an approximate measure of variance explained [69]. Although model selection and frequentist hypothesis testing should generally not be combined [66], we present *p*-values for readers accustomed to their presentation, but focus our discussion around variables retained during model selection.

Further details of the methodology used in this study can be found in electronic supplementary material appendix S1—supplementary methods. Data and code are available from https://doi.org/10.5061/dryad.1zcrjdfwr.

## 3. Results

We recorded 776 plant–pollinator interaction events involving 160 unique links (pairwise interactions) between 53 bee species and 56 plant species (electronic supplementary material, figure S1 and tables S5 and S6) across the 23 woodland remnants (figure 1). Each remnant network consisted of, on average, 6.8 (± 0.8 s.e.) bee species and 6.4 (± 0.5 s.e.) plant species (see more details in electronic supplementary material, appendix S2—supplementary results).

**Figure 2.**
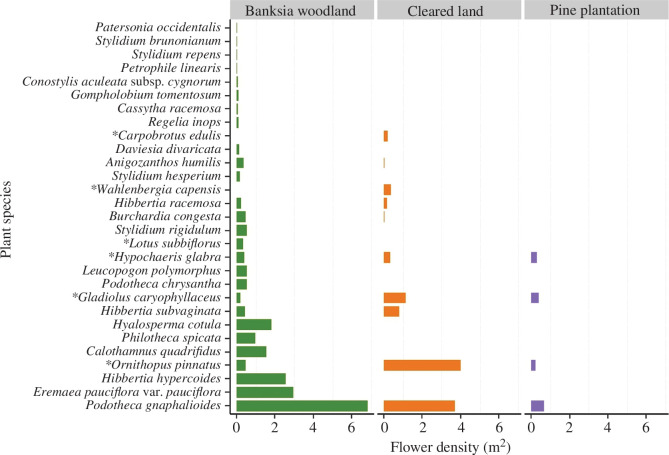
The most abundant partner plant species within each land-use type in the study area. Flower density per unit area (no. flowers m^−2^) of plant species in banksia woodland (green), cleared land (orange), and the non-native pine plantation (purple). Species with flower density values lower than 0.01 flowers m^−2^ are not shown. Non-native species are marked with an asterisk.

Across all three land-use types, we found that flower counts were dominated by a small number of species, with a long distribution tail of species with very low counts (less than 0.05 flowers m^−2^; figure 2). Among land uses, the native banksia woodland had a higher total number of plant species (56) and higher flower density per species per unit area (mean flower density m^−2^ = 0.39) than cleared land (richness = 30, mean flower density m^−2^ = 0.36) and pine plantation (richness = 18, mean flower density m^−2^ = 0.09; see more details in electronic supplementary material, appendix S2).

**Figure 3.**
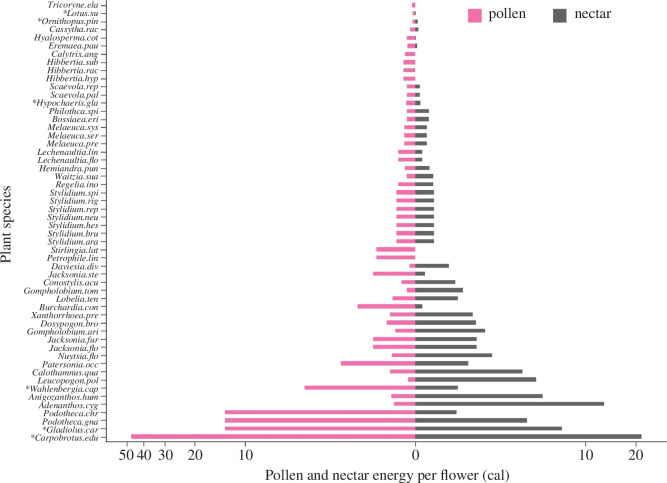
Relative contribution of pollen energy versus nectar energy to estimated total energy per flower per partner species (calories per flower). The plot is in logarithmic scale (log_10_(x)) and excludes two species where energy was reported only per inflorescence: *Banksia attenuata* (314 cal inflorescence^−1^) and *Banksia sessilis* var. *sessilis* (230 cal inflorescence^−1^) (see electronic supplementary material, table S4). Non-native species are marked with an asterisk. For full species names see electronic supplementary material, table S6.

**Figure 4.**
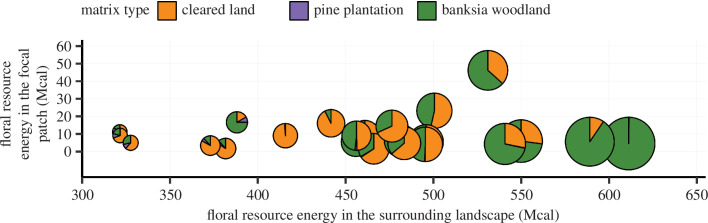
Estimated floral resource energy (mega calories, Mcal) of partner plant species to bees available in woodland patches (*y*-axis) and surrounding matrix land uses (*x*-axis). Data include estimated pollen and nectar energy of all partner species to bees at the patch- and landscape-level (1 km radius) in the 23 landscapes and are presented with sites in ascending order of total landscape resources. Data were scaled relative to mean landscape area for the *x*-axis, while total patch energy was plotted on the *y*-axis. Pie size s scaled relative to the total matrix floral resource energy.

Nectar (fructose, glucose and sucrose mass and energy content; electronic supplementary material, table S4) and pollen (pollen mass and energy content; electronic supplementary material, table S4) resource estimates per flower varied widely across the 56 plant species surveyed (see more details in electronic supplementary material appendix S2). We found a strong correlation between the pollen energy and nectar energy per flower (*r* = 0.79, *p* <0.0001; figure 3). The total floral resource energy (pollen + nectar) of plant partners of bees available in the surrounding matrix was much higher than that available within patch, with a high variation in floral resource energy in the different matrix land uses (figure 4).

**Figure 5.**
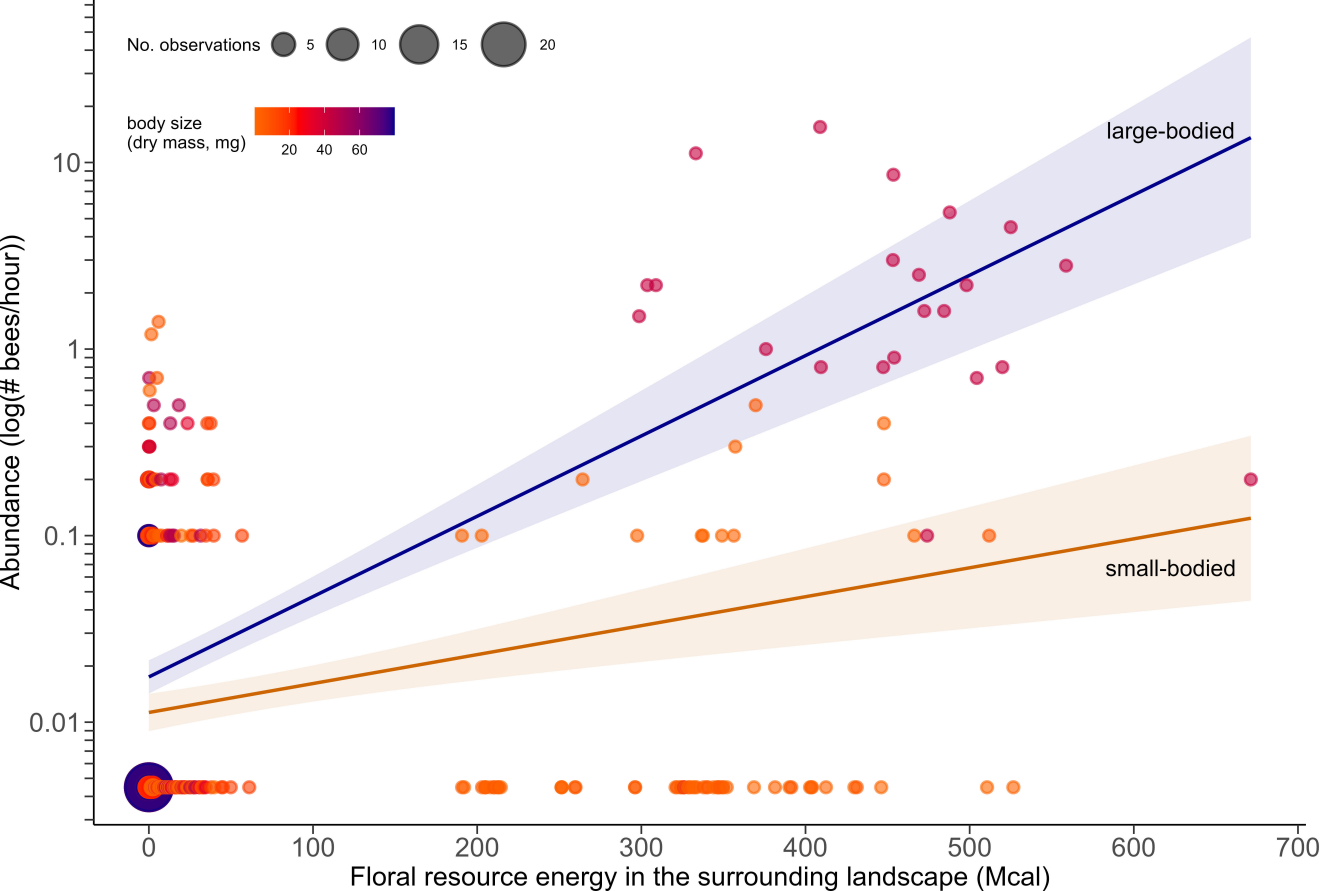
Abundance (log) of small-bodied (orange fitted line) and large-bodied (blue fitted line) bees in woodland remnants in response to increasing floral resource energy (energy in mega calories, Mcal) of interaction-partner plants in the surrounding landscape. Points represent capture rates per species per site (53 bee species at 23 sites), and fitted lines for small and large bees are model predictions from the best-fit GLMM, presented as the 5th and 95th percentiles of body mass (mg), respectively. Points and fitted lines are coloured according to bee body mass (estimated dry mass, mg), and point size reflects the number of overlapping data observations. Models were fitted with log-transformed body mass but are back-transformed to a linear scale for illustrative purposes.

In the GLMM model fitting, there were three models with equivalent explanatory power in the top-ranking model set (i.e. within 2 AICc units of the lowest AICc model; electronic supplementary material, table S7). Of these, the ‘best model’ (the most parsimonious model with the fewest parameters) had a significant effect of surrounding landscape floral resource energy on bee abundance (partially standardized effect = 1.313 [± 0.243], *p* < 0.001), translating to an increase in mean capture rates across the full range of energy (700 Mcal) of 8.0% of the total range of observed capture rates, irrespective of bee body size (held constant at mean). However, this influence of landscape-level floral resource energy also significantly varied with bee body size (partially standardized interactive effect = 1.235 [± 0.481] *p* < 0.01; electronic supplementary material, table S8, figure 5), with a substantial increase in mean capture rates for larger-bodied bees (standardized mean body size + 1 s.d.) equating to 87.0% of observed rates. In contrast, smaller bees (standardized mean body size – 1 s.d.) increased only by 0.7% of observed rates across the same resource gradient (figure 5). This modelled interaction of landscape-level floral resource energy and body size collectively accounted for 10.6% of the variance in bee capture rates, with the random effect of species explaining an additional 10.0% (electronic supplementary material, table S8). No effects of patch area, patch floral resource energy, or the interaction of body mass and patch floral resource energy were observed in models, suggesting that there was no strong evidence for our hypothesis that small-bodied bees would be more influenced by patch-level resource availability than larger-bodied bees.

## 4. Discussion

We hypothesized that energy availability of flowers within woodland remnants and in the surrounding landscape would increase abundance of their pollinators (bees) in remnant woodland patches. Secondly, we expected a shift in the relative importance of floral resources, from those within remnants to those in the surrounding landscape, as bee body size increases. Consistent with the first hypothesis, we found that bee population sizes in woodland patches increased with increasing floral energy resources in the surrounding matrix, though interestingly, there was no effect of within-patch floral resources. This finding implies that bees have wider foraging ranges than the local remnant [37] and that limitations of floral resources in the surrounding matrix, not the remnant patch, are most critical for the maintenance of bee communities in fragmented landscapes [24–26]. Bees entirely depend on flowers (especially nectar and pollen) for nutrition [47], and offspring production is greatly affected by limitation of foraging resources [22,70,71]. Native banksia woodland was the land cover type with the highest plant species richness and flower density per unit area, and therefore, we expected resource availability in native remnants to be the most important component of landscape resources and to drive bee abundance [23]. It is possible that, by showing a low range of variation in resource availability from remnant to remnant compared with the surrounding landscape (figure 4), that the range of resource availability within remnants was too limited to detect an effect. At the same time, we cannot rule out the possibility that a larger sample size would have detected additional patch-level effects, over and above landscape-scale influences. This weak within-patch effect of floral resources likely does not reflect other situations in which patches might vary dramatically in resource availability. In contrast, there was a large range of variation in floral resource availability in the surrounding matrix across sites (several orders of magnitude, figure 4), highlighting the essential role of floral resources in both remnant habitat and surrounding matrix for the persistence of bee populations in fragmented landscapes.

Consistent with our second hypothesis, energy resources in the surrounding matrix benefitted large-bodied bee species more than small-bodied bees (figure 5). Larger bees have higher energetic requirements [27] and would be able to persist only in landscapes where these higher energetic requirements are met [72], that is, where both remnant habitats and surrounding landscapes have sufficient floral resources. Larger bees can also fly further [35], which should allow them to adjust their foraging ranges to exploit landscape resource availability [36,37]. Our results, therefore, provide a mechanism (i.e. availability of energy rewards from partner plant species) to explain previously observed patterns such as a stronger negative response of large-bodied bees to agricultural intensification at the landscape-scale [73] and the greater dependence of smaller bees on large habitat patches [74]—presumably because they cannot access resources in the surrounding landscape. Our findings thus indicate that larger bee species can be particularly sensitive to decreasing floral resources at the landscape-level due to land use change [38], and should consequently benefit from restoration of floral resources in intensely modified landscapes, similarly to less mobile smaller bees [18].

In recent years, several studies have highlighted floral resource limitation due to land use change as a major driver of pollinator declines [11,13,75,76]. Our study is, however, one of the first to directly measure plant nectar and pollen energy resources at the landscape-level in a fragmented natural system and to relate this to the specific diet of individual pollinator species. The availability of floral energy rewards varied dramatically among land cover types surrounding native habitat patches, with remnant banksia woodland showing the highest richness of plant species and highest flower density per plant species per unit area. In contrast, non-native pine plantations were particularly resource-poor for bees, with a very low flower density per unit area and dominated by non-native plant species, which our surveys found were rarely utilized by native bees (see electronic supplementary material appendix S2—supplementary results, and electronic supplementary material, figure S1). Several plant species were only found in banksia woodlands (figure 2), highlighting the importance of remnant habitat as a source of floral rewards to meet different bee species’ energetic requirements. Conversely, plant species in cleared areas consisted mostly of non-native species and regeneration of native ephemeral and perennial forbs and shrubs, such as *Podotheca* spp. and *Hibbertia* spp. This was especially so in areas that had been clear-felled and burned since germination of several native species is induced by smoke [77,78]. Taken together, these findings indicate a detrimental effect of land clearing and replacement of native habitat by timber plantations on floral resource availability for bees at the landscape-level, whereby preferred plant species are lost and the matrix becomes increasingly resource-poor.

Energy rewards for bees per individual flower (nectar sugars and pollen mass) varied greatly among plant species (electronic supplementary material, table S4). This was expected given the high diversity of plant species in the study region, comprising a wide range of plant families and floral traits associated with different pollinator functional groups (e.g. short- and long-tongued bees) [31,79,80]. Importantly, pollen energy and nectar energy per flower were strongly correlated (figure 3), and moreover, the relative contribution of pollen energy to total energy per flower was substantial (over and above nectar energy). This suggests that previous studies that traditionally considered nectar as a primary source of energy for pollinators [43,81,82] may have greatly underestimated total energy rewards per flower. Our study thus provides data on the important contribution of pollen, which should be considered when designing future resource assessment studies for pollinators.

It is noteworthy that flowers with the highest energy content were not necessarily visited by more bee species. This may reflect variation in pollinator energy demands since bee species have specific foraging requirements and floral rewards are tailored to meet these [30,72]. For example, the introduced South African plants *Carpobrotus edulis* (Aizoaceae) and *Gladiolus caryophillaceus* (Iridaceae) had the highest energy per flower but were visited almost entirely by the introduced honeybee, *Apis mellifera*, with the exception of one visit to *C. edulis* flowers by a native bee species (Megachile sp.2, Megachilidae; electronic supplementary material S1). Conversely, flower heads of the endemic *Podotheca gnaphalioides* and *Podotheca chrysantha* (Asteraceae) were third and fourth-ranked in energy produced per flower unit, but visited by several bee species. Flowers of a variety of species found almost exclusively in banksia woodlands, such as *Gompholobium tomentosum* (Fabaceae), *Gompholobium aristatum* (Fabaceae), *Lechenaultia floribunda* (Goodeniaceae), *Philotheca spicata* (Rutaceae) and *Regelia inops* (Myrtaceae), produced relatively less energy per flower, and were comparatively less abundant (figure 2). However, they were among the plant species visited by the highest number of bee species, potentially indicating that these are important ‘hub’ species in the system; that is, those with a high number of interspecific links that are visited disproportionally more than expected given their floral abundance. Bees select flowers on the basis of other nutritional rewards (e.g. amino acids) in addition to calories, as these rewards can influence fitness [83]. Here, we only focused on energy rather than other nutritional requirements, whereas preferences for other nutrients or floral traits may explain the residual variation generated by these plant species whose visitation rates do not relate to energy. Hub species are very important from a conservation point of view, as their loss could instigate wider disruption and potentially species loss through the plant–pollinator networks [84,85].

## 5. Conclusion

Floral resource limitation has been increasingly recognized as a primary driver of pollinator losses in response to land use change [13,14,43], and its impact depends on species-specific ecological and life-history traits related to the use and acquisition of these resources [86,87]. By assessing floral energy resources (comprising both nectar and pollen energy) of plant interaction partners at the landscape scale, we demonstrated that energetics of the surrounding landscape drive increased bee abundance in remnant natural habitat, while the greater observed response of large-bodied bees indicates their greater reliance on landscape-level resources. This increased sensitivity of larger bees to reduced floral resources in the surrounding matrix thus provides a mechanism by which land use change causes bee species declines and declining mean body size within isolated habitat remnants in modified landscapes [13,88–90]. Our work contributes to a growing knowledge base on the effects of land use change on pollinator declines mediated by floral resource losses [12,76,91,92], pointing to important moderating effects of the availability of landscape-level floral resources on the persistence of bee assemblages in remnant habitat, which have crucial consequences for the conservation of pollinators and essential pollination services in human-modified landscapes.

## Data Availability

Data and R code used in this study are available from Dryad [93]. Supplementary material is available online [94].

## References

[1] Wagner DL. 2020 Insect declines in the anthropocene. Annu. Rev. Entomol. **65**, 457–480. (10.1146/annurev-ento-011019-025151) 31610138

[2] Newbold T *et al*. 2015 Global effects of land use on local terrestrial biodiversity. Nature **520**, 45–50. (10.1038/nature14324) 25832402

[3] Seto KC, Güneralp B, Hutyra LR. 2012 Global forecasts of urban expansion to 2030 and direct impacts on biodiversity and carbon pools. Proc. Natl Acad. Sci. USA **109**, 16083–16088. (10.1073/pnas.1211658109) 22988086 PMC3479537

[4] Ewers RM, Didham RK. 2006 Confounding factors in the detection of species responses to habitat fragmentation. Biol. Rev. Camb. Philos. Soc. **81**, 117–142. (10.1017/S1464793105006949) 16318651

[5] Driscoll DA, Banks SC, Barton PS, Lindenmayer DB, Smith AL. 2013 Conceptual domain of the matrix in fragmented landscapes. Trends Ecol. Evol. (Amst.) **28**, 605–613. (10.1016/j.tree.2013.06.010) 23883740

[6] Kremen C, Merenlender AM. 2018 Landscapes that work for biodiversity and people. Science **362**, eaau6020. (10.1126/science.aau6020) 30337381

[7] Potts SG, Biesmeijer JC, Kremen C, Neumann P, Schweiger O, Kunin WE. 2010 Global pollinator declines: trends, impacts and drivers. Trends Ecol. Evol. (Amst.) **25**, 345–353. (10.1016/j.tree.2010.01.007) 20188434

[8] Winfree R, Bartomeus I, Cariveau DP. 2011 Native pollinators in anthropogenic habitats. Annu. Rev. Ecol. Evol. Syst. **42**, 1–22. (10.1146/annurev-ecolsys-102710-145042)

[9] Stout JC, Tiedeken EJ. 2017 Direct interactions between invasive plants and native pollinators: evidence, impacts and approaches. Funct. Ecol. **31**, 38–46. (10.1111/1365-2435.12751)

[10] Bartomeus I, Fründ J, Williams NM. 2016 Invasive plants as novel food resources, the pollinators’ perspective. In Biological invasions and animal behaviour (eds J Weis, D Sol), pp. 119–132. Cambridge, UK: Cambridge University Press. (10.1017/CBO9781139939492)

[11] Roulston TH, Goodell K. 2011 The role of resources and risks in regulating wild bee populations. Annu. Rev. Entomol. **56**, 293–312. (10.1146/annurev-ento-120709-144802) 20822447

[12] Goulson D, Nicholls E, Botías C, Rotheray EL. 2015 Bee declines driven by combined stress from parasites, pesticides, and lack of flowers. Science **347**, 1255957. (10.1126/science.1255957) 25721506

[13] Scheper J, Reemer M, van Kats R, Ozinga WA, van der Linden GTJ, Schaminée JHJ, Siepel H, Kleijn D. 2014 Museum specimens reveal loss of pollen host plants as key factor driving wild bee decline in The Netherlands. Proc. Natl Acad. Sci. USA **111**, 17552–17557. (10.1073/pnas.1412973111) 25422416 PMC4267333

[14] Biesmeijer JC *et al*. 2006 Parallel declines in pollinators and insect-pollinated plants in Britain and the Netherlands. Science **313**, 351–354. (10.1126/science.1127863) 16857940

[15] Papanikolaou AD, Kühn I, Frenzel M, Kuhlmann M, Poschlod P, Potts SG, Roberts SPM, Schweiger O. 2017 Wild bee and floral diversity co‐vary in response to the direct and indirect impacts of land use. Ecosphere **8**, e02008. (10.1002/ecs2.2008)

[16] Bukovinszky T, Verheijen J, Zwerver S, Klop E, Biesmeijer JC, Wäckers FL, Prins HHT, Kleijn D. 2017 Exploring the relationships between landscape complexity, wild bee species richness and reproduction, and pollination services along a complexity gradient in the Netherlands. Biol. Conserv. **214**, 312–319. (10.1016/j.biocon.2017.08.027)

[17] Cole LJ, Brocklehurst S, Robertson D, Harrison W, McCracken DI. 2017 Exploring the interactions between resource availability and the utilisation of semi-natural habitats by insect pollinators in an intensive agricultural landscape. Agric. Ecosyst. Environ. **246**, 157–167. (10.1016/j.agee.2017.05.007)

[18] Kremen C, M’Gonigle LK. 2015 Small‐scale restoration in intensive agricultural landscapes supports more specialized and less mobile pollinator species. J. Appl. Ecol. **52**, 602–610. (10.1111/1365-2664.12418)

[19] Scheper J *et al*. 2015 Local and landscape‐level floral resources explain effects of wildflower strips on wild bees across four European countries. J. Appl. Ecol. **52**, 1165–1175. (10.1111/1365-2664.12479)

[20] Blaauw BR, Isaacs R. 2014 Flower plantings increase wild bee abundance and the pollination services provided to a pollination‐dependent crop. J. Appl. Ecol. **51**, 890–898. (10.1111/1365-2664.12257)

[21] Häussler J, Sahlin U, Baey C, Smith HG, Clough Y. 2017 Pollinator population size and pollination ecosystem service responses to enhancing floral and nesting resources. Ecol. Evol. **7**, 1898–1908. (10.1002/ece3.2765) 28331597 PMC5355185

[22] Kremen C *et al*. 2007 Pollination and other ecosystem services produced by mobile organisms: a conceptual framework for the effects of land‐use change. Ecol. Lett. **10**, 299–314. (10.1111/j.1461-0248.2007.01018.x) 17355569

[23] Garibaldi LA *et al*. 2011 Stability of pollination services decreases with isolation from natural areas despite honey bee visits. Ecol. Lett. **14**, 1062–1072. (10.1111/j.1461-0248.2011.01669.x) 21806746

[24] Williams NM, Regetz J, Kremen C. 2012 Landscape-scale resources promote colony growth but not reproductive performance of bumble bees. Ecology **93**, 1049–1058. (10.1890/11-1006.1) 22764491

[25] Kennedy CM *et al*. 2013 A global quantitative synthesis of local and landscape effects on wild bee pollinators in agroecosystems. Ecol. Lett. **16**, 584–599. (10.1111/ele.12082) 23489285

[26] Mallinger RE, Gibbs J, Gratton C. 2016 Diverse landscapes have a higher abundance and species richness of spring wild bees by providing complementary floral resources over bees’ foraging periods. Landsc. Ecol. **31**, 1523–1535. (10.1007/s10980-015-0332-z)

[27] McCallum KP, McDougall FO, Seymour RS. 2013 A review of the energetics of pollination biology. J. Comp. Physiol. B, Biochem. Syst. Environ. Physiol. **183**, 867–876. (10.1007/s00360-013-0760-5) 23653068

[28] Brown JH, Calder WA, Kodric-brown A. 1978 Correlates and consequences of body size in nectar-feeding birds. Am. Zool. **18**, 687–738. (10.1093/icb/18.4.687)

[29] Duell ME, Klok CJ, Roubik DW, Harrison JF. 2022 Size-dependent scaling of stingless bee flight metabolism reveals an energetic benefit to small body size. Integr. Comp. Biol. **62**, 1429–1438. (10.1093/icb/icac131) 36066644 PMC9825317

[30] Heinrich B, Raven PH. 1972 Energetics and pollination ecology. Science **176**, 597–602. (10.1126/science.176.4035.597) 17778157

[31] Fenster CB, Armbruster WS, Wilson P, Dudash MR, Thomson JD. 2004 Pollination syndromes and floral specialization. Annu. Rev. Ecol. Evol. Syst. **35**, 375–403. (10.1146/annurev.ecolsys.34.011802.132347)

[32] Petanidou T, Vokou D. 1990 Pollination and pollen energetics in mediterranean ecosystems. Am. J. Bot. **77**, 986–992. (10.1002/j.1537-2197.1990.tb13593.x)

[33] Balfour NJ, Shackleton K, Arscott NA, Roll-Baldwin K, Bracuti A, Toselli G, Ratnieks FLW. 2021 Energetic efficiency of foraging mediates bee niche partitioning. Ecology **102**, e03285. (10.1002/ecy.3285) 33462847

[34] Harrison JF, Roberts SP. 2000 Flight respiration and energetics. Annu. Rev. Physiol. **62**, 179–205. (10.1146/annurev.physiol.62.1.179) 10845089

[35] Greenleaf SS, Williams NM, Winfree R, Kremen C. 2007 Bee foraging ranges and their relationship to body size. Oecologia **153**, 589–596. (10.1007/s00442-007-0752-9) 17483965

[36] Steffan-Dewenter I, Kuhn A. 2003 Honeybee foraging in differentially structured landscapes. Proc. R. Soc. Lond. B **270**, 569–575. (10.1098/rspb.2002.2292) PMC1691282 12769455

[37] Carvell C, Jordan WC, Bourke AFG, Pickles R, Redhead JW, Heard MS. 2012 Molecular and spatial analyses reveal links between colony‐specific foraging distance and landscape‐level resource availability in two bumblebee species. Oikos **121**, 734–742. (10.1111/j.1600-0706.2011.19832.x)

[38] Wray JC, Neame LA, Elle E. 2014 Floral resources, body size, and surrounding landscape influence bee community assemblages in oak‐savannah fragments. Ecol. Entomol. **39**, 83–93. (10.1111/een.12070)

[39] Bennett AB, Lovell S. 2019 Landscape and local site variables differentially influence pollinators and pollination services in urban agricultural sites. PLoS One **14**, e0212034, (10.1371/journal.pone.0212034) 30759171 PMC6373950

[40] Weiner CN, Werner M, Linsenmair KE, Blüthgen N. 2014 Land-use impacts on plant–pollinator networks: interaction strength and specialization predict pollinator declines. Ecology **95**, 466–474. (10.1890/13-0436.1) 24669739

[41] Eklöf A *et al*. 2013 The dimensionality of ecological networks. Ecol. Lett. **16**, 577–583. (10.1111/ele.12081) 23438174

[42] Peralta G, Webber CJ, Perry GLW, Stouffer DB, Vázquez DP, Tylianakis JM. 2023 Scale‐dependent effects of landscape structure on pollinator traits, species interactions and pollination success. Ecography **2023**. (10.1111/ecog.06453)

[43] Baude M, Kunin WE, Boatman ND, Conyers S, Davies N, Gillespie MAK, Morton RD, Smart SM, Memmott J. 2016 Historical nectar assessment reveals the fall and rise of floral resources in Britain. Nature **530**, 85–88. (10.1038/nature16532) 26842058 PMC4756436

[44] Hicks DM *et al*. 2016 Food for pollinators: quantifying the nectar and pollen resources of urban flower meadows. PLoS One **11**, e0158117. (10.1371/journal.pone.0158117) 27341588 PMC4920406

[45] Ausseil AGE, Dymond JR, Newstrom L. 2018 Mapping floral resources for honey bees in New Zealand at the catchment scale. Ecol. Appl. **28**, 1182–1196. (10.1002/eap.1717) 29528528

[46] Potts SG, Vulliamy B, Dafni A, Ne’eman G, Willmer P. 2003 Linking bees and flowers: how do floral communities structure pollinator communities? Ecology **84**, 2628–2642. (10.1890/02-0136)

[47] Michener CD. The bees of the world, 2nd edn. Baltimore, MD: The Johns Hopkins University Press. (10.56021/9780801885730). See https://www.press.jhu.edu/books/title/9040/bees-world.

[48] Hopper SD, Gioia P. 2004 The Southwest Australian floristic region: evolution and conservation of a global hot spot of biodiversity. Annu. Rev. Ecol. Evol. Syst. **35**, 623–650. (10.1146/annurev.ecolsys.35.112202.130201)

[49] Department of the Environment and Energy AG. 2016 Banksia woodlands of the swan Coastal plain: a nationally protected ecological community. pp. 1–20. See https://www.environment.gov.au/system/files/resources/8ed3311d-55c1-45a8-b240-63a5663c2fea/files/banksia-woodlands-scp-guide.pdf (accessed 16 April 2021).

[50] Kendall L. 2018 package ‘Pollimetry’: estimate Pollinator body size and Co-varying ecological Traits. pp. 1–10. See https://CRAN.R-project.org/package=pollimetry.

[51] Kendall LK *et al*. 2019 Pollinator size and its consequences: robust estimates of body size in pollinating insects. Ecol. Evol. **9**, 1702–1714. (10.1002/ece3.4835) 30847066 PMC6392396

[52] Shreeves G, Field J. 2008 Parental care and sexual size dimorphism in wasps and bees. Behav. Ecol. Sociobiol. **62**, 843–852. (10.1007/s00265-007-0510-3)

[53] QGIS Development Team. 2018 QGIS geographic information system. See http://qgis.osgeo.org.

[54] Carvalheiro LG *et al*. 2014 The potential for indirect effects between co-flowering plants via shared pollinators depends on resource abundance, accessibility and relatedness. Ecol. Lett. **17**, 1389–1399. (10.1111/ele.12342) 25167890

[55] Corbet SA. 2003 Nectar sugar content: estimating standing crop and secretion rate in the field. Apidologie (Celle) **34**, 1–10. (10.1051/apido:2002049)

[56] Morrant DS, Schumann R, Petit S. 2009 Field methods for sampling and storing nectar from flowers with low nectar volumes. Ann. Bot. **103**, 533–542. (10.1093/aob/mcn241) 19074446 PMC2707336

[57] Nicolson SW, Thornburg RW. 2007 Nectar chemistry. In Nectaries and nectar (eds SW Nicolson, M Nepi, E Pacini), pp. 215–264. Dordrecht, The Netherlands: Springer. (10.1007/978-1-4020-5937-7)

[58] Zwolinski BJ, Wilhoit RC. 1972 Heats of formation and heats of combustion. In American Institute of Physics handbook (ed. DE Gray), p. 334. New York, NY: McGraw-Hill.

[59] R Core Team. 2023 R: a language and environment for statistical computing. Vienna, Austria: R Foundation for Statistical Computing. See https://www.r-project.org/.

[60] Brooks ME, Kristensen K, van Benthem KJ, Magnusson A, Berg CW, Nielsen A, Skaug HJ, Mächler M, Bolker BM. 2017 glmmTMB balances speed and flexibility among packages for zero-inflated generalized linear mixed modeling. R J. **9**, 378–400. (10.32614/RJ-2017-066)

[61] Magnusson A, Skaug H, Nielsen A, Berg C, Kristensen K, Maechler M, Sadat N, van Bentham K. 2019 Package ‘glmmTMB’: generalized linear mixed models using template model builder. See https://cran.r-project.org/web/packages/glmmTMB/.

[62] Mazerolle M. 2019 AICcmodavg: model selection and multimodel inference based on (Q)AIC(c). See https://cran.r-project.org/web/packages/AICcmodavg/AICcmodavg.pdf.

[63] Gelman A. 2018 Package ‘arm’: data analysis using regression and multilevel/hierarchicalmodels. See https://cran.r-project.org/web/packages/arm/index.html.

[64] Gelman A. 2008 Scaling regression inputs by dividing by two standard deviations. Stat. Med. **27**, 2865–2873. (10.1002/sim.3107) 17960576

[65] Zuur AF, Ieno EN, Elphick CS. 2010 A protocol for data exploration to avoid common statistical problems. Methods Ecol. Evol. **1**, 3–14. (10.1111/j.2041-210X.2009.00001.x)

[66] Burnham KP, Anderson DR. 2002 Model selection and multimodel inference: a practical information-theoretic approach, 2nd edn. New York, NY: Springer.

[67] Grueber CE, Nakagawa S, Laws RJ, Jamieson IG. 2011 Multimodel inference in ecology and evolution: challenges and solutions. J. Evol. Biol. **24**, 699–711. (10.1111/j.1420-9101.2010.02210.x) 21272107

[68] Lüdecke D, Makowski D, Waggoner P, Patil I. 2020 Package ‘performance’: assessment of regression models performance. See https://easystats.github.io/performance/.

[69] Nakagawa S, Johnson PCD, Schielzeth H. 2017 The coefficient of determination R^2^ and intra-class correlation coefficient from generalized linear mixed-effects models revisited and expanded. J. R. Soc. Interface **14**, 1–11, (10.1098/rsif.2017.0213) PMC5636267 28904005

[70] Renauld M, Hutchinson A, Loeb G, Poveda K, Connelly H. 2016 Landscape simplification constrains adult size in a native ground-nesting Bee. PLoS One **11**, e0150946, (10.1371/journal.pone.0150946) 26943127 PMC4778946

[71] Bosch J. 2008 Production of undersized offspring in a solitary bee. Anim. Behav. **75**, 809–816. (10.1016/j.anbehav.2007.06.018)

[72] Heinrich B. 1975 Energetics of pollination. Annu. Rev. Ecol. Syst. **6**, 139–170. (10.1146/annurev.es.06.110175.001035)

[73] Benjamin FE, Reilly JR, Winfree R. 2014 Pollinator body size mediates the scale at which land use drives crop pollination services. J. Appl. Ecol. **51**, 440–449. (10.1111/1365-2664.12198)

[74] Jauker B, Krauss J, Jauker F, Steffan-Dewenter I. 2013 Linking life history traits to pollinator loss in fragmented calcareous grasslands. Landsc. Ecol. **28**, 107–120. (10.1007/s10980-012-9820-6)

[75] Vaudo AD, Tooker JF, Grozinger CM, Patch HM. 2015 Bee nutrition and floral resource restoration. Curr. Opin. Insect Sci. **10**, 133–141, (10.1016/j.cois.2015.05.008) 29588000

[76] Vanbergen AJ, Initiative the IP. 2013 Threats to an ecosystem service: pressures on pollinators. Front. Ecol. Environ. **11**, 251–259. (10.1890/120126)

[77] Dixon KW, Roche S, Pate JS. 1995 The promotive effect of smoke derived from burnt native vegetation on seed germination of Western Australian plants. Oecologia **101**, 185–192. (10.1007/BF00317282) 28306789

[78] Rokich DP, Dixon KW, Sivasithamparam K, Meney KA. 2002 Smoke, mulch, and seed broadcasting effects on woodland restoration in Western Australia. Restor. Ecol. **10**, 185–194. (10.1046/j.1526-100X.2002.02040.x)

[79] Dupont YL, Hansen DM, Rasmussen JT, Olesen JM. 2004 Evolutionary changes in nectar sugar composition associated with switches between bird and insect pollination: the Canarian bird–flower element revisited. Funct. Ecol. **18**, 670–676. (10.1111/j.0269-8463.2004.00891.x)

[80] Parachnowitsch AL, Manson JS, Sletvold N. 2019 Evolutionary ecology of nectar. Ann. Bot. **123**, 247–261. (10.1093/aob/mcy132) 30032269 PMC6344224

[81] Petanidou T. 2005 Sugars in mediterranean floral nectars: an ecological and evolutionary approach. J. Chem. Ecol. **31**, 1065–1088. (10.1007/s10886-005-4248-y) 16124233

[82] Chalcoff VR, Gleiser G, Ezcurra C, Aizen MA. 2017 Pollinator type and secondarily climate are related to nectar sugar composition across the angiosperms. Evol. Ecol. **31**, 585–602. (10.1007/s10682-017-9887-2)

[83] Hoover SER, Ladley JJ, Shchepetkina AA, Tisch M, Gieseg SP, Tylianakis JM. 2012 Warming, CO_2_, and nitrogen deposition interactively affect a plant–pollinator mutualism. Ecol. Lett. **15**, 227–234. (10.1111/j.1461-0248.2011.01729.x) 22221802

[84] Olesen JM, Bascompte J, Dupont YL, Jordano P. 2007 The modularity of pollination networks. Proc. Natl Acad. Sci. USA **104**, 19891–19896. (10.1073/pnas.0706375104) 18056808 PMC2148393

[85] Biella P, Akter A, Ollerton J, Tarrant S, Janeček Š, Jersáková J, Klečka J. 2018 Experimental loss of generalist plants reveals alterations in plant–pollinator interactions and a constrained flexibility of foraging. bioRxiv 279430. (10.1101/279430) PMC6517441 31089144

[86] Williams NM, Crone EE, Roulston TH, Minckley RL, Packer L, Potts SG. 2010 Ecological and life-history traits predict bee species responses to environmental disturbances. Biol. Conserv. **143**, 2280–2291. (10.1016/j.biocon.2010.03.024)

[87] Jauker B, Krauss J, Jauker F, Steffan-Dewenter I. 2013 Linking life history traits to pollinator loss in fragmented calcareous grasslands. Landsc. Ecol. **28**, 107–120. (10.1007/s10980-012-9820-6)

[88] Rader R, Bartomeus I, Tylianakis JM, Laliberté E. 2014 The winners and losers of land use intensification: pollinator community disassembly is non‐random and alters functional diversity. Divers. Distrib. **20**, 908–917. (10.1111/ddi.12221)

[89] Bommarco R, Biesmeijer JC, Meyer B, Potts SG, Pöyry J, Roberts SPM, Steffan-Dewenter I, Ockinger E. 2010 Dispersal capacity and diet breadth modify the response of wild bees to habitat loss. Proc. R. Soc. B. **277**, 2075–2082. (10.1098/rspb.2009.2221) PMC2880091 20219735

[90] Bartomeus I, Ascher JS, Gibbs J, Danforth BN, Wagner DL, Hedtke SM, Winfree R. 2013 Historical changes in northeastern US bee pollinators related to shared ecological traits. Proc. Natl Acad. Sci. USA **110**, 4656–4660. (10.1073/pnas.1218503110) 23487768 PMC3606985

[91] Carvell C, Roy DB, Smart SM, Pywell RF, Preston CD, Goulson D. 2006 Declines in forage availability for bumblebees at a national scale. Biol. Conserv. **132**, 481–489. (10.1016/j.biocon.2006.05.008)

[92] Kleijn D, Raemakers I. 2008 A retrospective analysis of pollen host plant use by stable and declining bumble bee species. Ecology **89**, 1811–1823. (10.1890/07-1275.1) 18705369

[93] Pille Arnold J, Tylianakis JM, Murphy MV, Cawthray GR, Webber BL, Didham RK. 2023 Data from: Body-size-dependent effects of landscape-level resource energetics on pollinator abundance in woodland remnants. Dryad Digital Repository. (10.5061/dryad.1zcrjdfwr) PMC11286198 38864334

[94] Pille Arnold J, Tylianakis JM, Murphy MV, Cawthray GR, Webber BL, Didham RK. 2024 Supplementary material from: Body-size-dependent effects of landscape-level resource energetics on pollinator abundance in woodland remnants. Figshare. (10.6084/m9.figshare.c.7227085) PMC11286198 38864334

